# Stress ulcer prophylaxis in critically ill poisoned patients

**DOI:** 10.1186/2008-2231-20-80

**Published:** 2012-11-27

**Authors:** Omid Mehrpour, Bita Dadpour, Nasim Zamani

**Affiliations:** 1 Medical Toxicology and Drug Abuse Research Center (MTDRC), Birjand, University of Medical Science, Pasdaran Avenue, Birjand, Iran; 2 Addiction Research Centre, Mashhad University of Medial Toxicology, Mashhad, Iran; 3 Department of Clinical Toxicology, Loghman Hakim Hospital, Shahid Beheshti University of Medical Sciences, Tehran, Iran

## Letter to the editor

Management of critically ill patients in the intensive care unit (ICU) is an important issue in medicine [1,2]. These patients may have clinically important bleeding, which is associated with an increased mortality rate. Mechanical ventilation may also increase the risk of bleeding in the upper gastrointestinal system. Sucralfate, histamine-2-receptor antagonists, and proton pumps inhibitors (PPIs) are widely used for stress ulcer prophylaxis in critically ill adults [3]. Available data shows that PPIs are safe and efficacious for increasing intragastric pH in critically ill patients [4]. Since the superiority of the PPIs over histamine-2-receptor antagonists or sucralfate is not yet proved, they should only be used as an alternative to these agents.

Poisoned patients may need to be admitted in ICU because of their critical condition. Poisoning with fatal agents such as aluminum phosphide, paraquat, organophosphate, or massive pharmaceutical drug overdoses usually needs ICU care and sometimes mechanical ventilation [5,6]. A question is therefore arisen: which of these agents is better to be used for ulcer prophylaxis in poisoned patients?

Affection of the cytochrome P450 (CYP) by toxins or pharmaceutical drugs is an important point in answering this question. Generally, most of the toxicants have a liver metabolism [7]. The number of the known P450 enzyme inducers or inhibitors exceeds 1000, but P450 3A4 is the most frequent CYP in the human liver and is known to metabolize the majority of drugs whose biotransformation is known [8]. So, inhibition or induction of CYP may increase or decrease the final metabolites of the toxicants and may have a value in the treatment of poisoned patients in the ICU.

Histamine-2-receptor antagonists are known as CYP inhibitors [9] and PPIs are inducers of CYP [8]. PPIs can therefore be recommended for the intoxications in which a CYP inhibitor is involved (such as antidepressant intoxication) or the medications which are toxic in origin but induce non-toxic metabolites. Histamine-2 receptor antagonists may be more useful in the poisonings with CYP inducers (such as carbamazepine or Phenobarbital toxicities) or in cases whose metabolits are more toxic than the parent medication (such as acetaminophen, tramadol, etc.). This may affect the patients’ prognosis as well as their hospital stay. We, therefore, would like to encourage toxicologists to provide studies to evaluate such potential benefits of each type of these ulcer protectors in each poisoning.

## Competing interests

The authors declare that they have no competing interests.

## Authors’ contributions

BD and NZ did bibliography and drafted the article. OM gave the idea and completed/edited/revised the article. All authors read and approved the final manuscript.
